# Road safety in low- and middle-income countries: Role of police strike

**DOI:** 10.7189/jogh.12.03070

**Published:** 2022-11-08

**Authors:** Kehinde O Ogunyemi, David O Alao, Eteete M Adam, Chenchita Malolan, Oluwole Olaomi

**Affiliations:** 1 Department of Community Medicine, Babcock University Teaching Hospital, Babcock University, Ilishan-Remo, Ogun State, Nigeria; 2 Hubert Department of Global Health, Rollins School of Public Health, Emory University, Atlanta, Georgia, USA; 3 Department of Political Science and Public Administration, Babcock University, Ilishan-Remo, Ogun State, Nigeria; 4 Department of Jurispudence and Private Law, Babcock University, Ilishan-Remo, Ogun State, Nigeria; 5 Office of Global Health, Department of Surgery, University of Texas Southwestern Medical Center, Dallas, Texas, USA; 6 Department of Surgery, National Trauma Centre, National Hospital Abuja, Abuja, Federal Capital Territory, Nigeria

Road traffic injuries (RTIs) constitute a significant global health burden responsible for 1.35 million deaths and 50 million disabilities annually [1]. Also, RTIs are the leading cause of death among children and young adults aged 5-29 years, worldwide [1]. Low- and middle-income countries (LMICs) continue to be disproportionately impacted by the burden of RTIs and deaths [1]. Currently, the proportion of RTIs and deaths in LMICs is estimated at over 90% of the global burden and projected to further increase by 80% over the next decade, unless necessary and timely actions are taken at national, and global levels [1].

Recognizing that road safety is a complex issue that involves sectors beyond health care such as transport and police, the international community frequently calls for a multidisciplinary perspective, particularly with a focus on social context, to improve the understanding of RTIs and its application in the reduction of associated mortality, and disability [1]. This recommendation remained one of the key highlights of the new Road Safety Global Decade of Action for 2021-2030 discussed during the recently concluded United Nations (UN) High-Level Meeting on Global Road Safety (June 30-July 1, 2022), with a vision of reducing the number of global RTIs and deaths by 50% by 2030 and ensuring sustainable transport as aligned with the Sustainable Development Goals (SDGs) target 3.6 and 11.2 respectively [2,3].

Police represent the major first responder population that provides pre-hospital trauma care for road traffic injury victims in LMICs [1]. Though this responsibility is embodied in the police statutory mandate to protect the lives of citizens, it is not uncommon for police worldwide to embark on strike action for multiple reasons such as poor salary and unsatisfactory housing conditions as documented in the literature [4-7]. In fact, evidence shows that low income–an important social determinant of health–remains one of the leading facilitators of police strikes globally [8]. While the public health impact of police strikes on road safety is poorly documented, we argue that a police strike has the capacity to threaten road safety. Specifically, police strikes could cause excess preventable road traffic deaths and contribute to an increased global road mortality burden by two synergistic mechanisms. These mechanisms include first, lack of or suboptimal first aid for road traffic injury victims from inaccessibility to general-duty police; and second, delayed access of road traffic injury victims to definitive health care facilities due to traffic congestion, partially resulting from unavailability of traffic police. Despite the possibility of a police strike, empirical data describing excess road traffic deaths associated with police strike is scarce with nothing known about global health advocacy on this issue.

## EQUITY LENS UNDERSTANDING OF ROAD SAFETY AND POLICE STRIKE INTERSECTION: A CASE STUDY OF NIGERIA

Understanding the intersection between road safety and police strike is particularly relevant in LMICs like Nigeria, where almost 50% of road traffic injury victims receive pre-hospital trauma care interventions from police [9]. And how the police strike may put the road traffic injury victims at a higher risk of death by lack of access to life-saving pre-hospital trauma care interventions or inappropriate care by untrained bystanders, given current evidence on low rate of bystander first aid in the setting [10]. Nonetheless, understanding the impact of police strike on road safety is of global health importance because it is an issue that is centred on human rights and equity principles.

Creating a balance between support and prevention of police strike for advocacy implications poses a great challenge. Further, this challenge would certainly require an evidence-based approach and contextual analysis due to differential risk factors and legal framework within and between countries. For example, in Nigeria, where there was a recent report of a nationwide police strike threat [11], the social determinants of health including low income and poor housing among police in addition to their health risks (eg, psychological distress), which suggest an unacceptably high level of job dissatisfaction, and inequity might be contributing to the strike threat [12,13].

**Figure:**
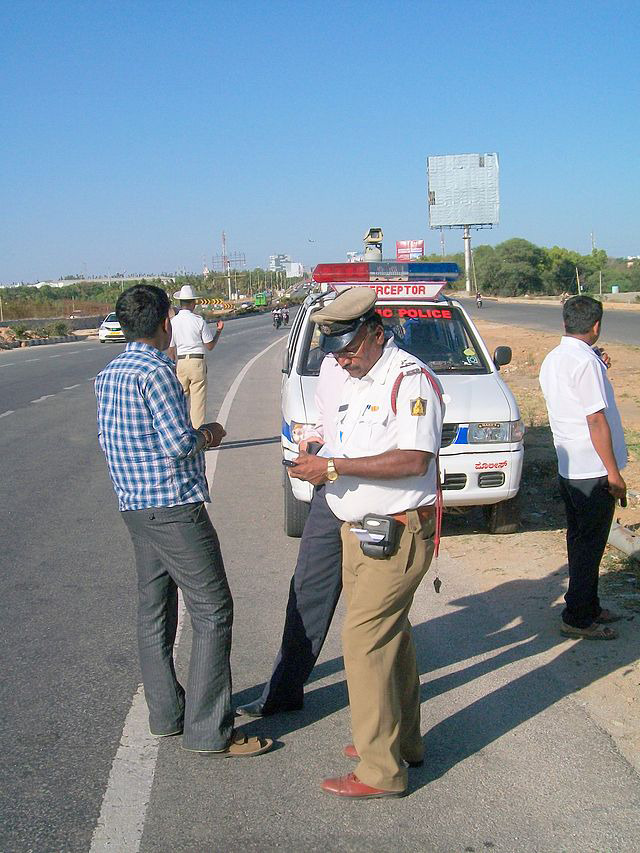
Photo: Traffic Sub-Inspector of Police of Bangalore Traffic Police with Interceptor using Black Berry.jpg. Source: Wikimedia Commons CC 0, in public domain https://en.m.wikipedia.org/wiki/File:Traffic_Sub-Inspector_of_Police_of_Bangalore_Traffic_Police_with_Interceptor_using_Black_Berry.jpg

**Figure 1:**
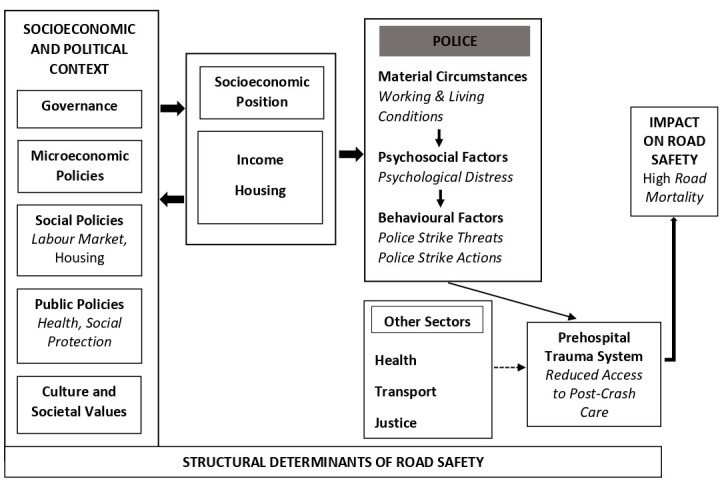
Adapted WHO Framework on Social Determinants of Health [14] for relationship between road safety and police strike.

Considering a previous police strike in Nigeria about two decades ago [5], with anecdotal evidence on several strike attempts and the demonstrated potential impact of police strikes on road safety, we argue that police strike should be considered as one of the wider structural determinants of road safety, especially in LMICs as presented in an adapted World Health Organization Framework on Social Determinants of Health in **Figure 1** [14]. Also, while global health may recognize the human rights of police to engage in a strike, absolute support for it may contribute to the widening of health inequity gap among citizens as well. This is because citizens who may be involved in road traffic crashes during police strikes, especially those with severe injuries will be disenfranchised to receive life-saving pre-hospital trauma care interventions. And by these considerations, we argue that the issue of police strike demands to be recognized by road safety and global health policy makers, as a potential barrier to achieving not just road safety goals but also SDGs, particularly those that seek to attain health for all, improved economic productivity, safe and sustainable communities, and peaceful social cohesion. Further, it is our expectation that this understanding as being extended by more research, would catalyse dialogues and strong political commitments at national and global levels for efficient policy actions to prevent police strikes, and its associated probable outcomes including but not limited to excess road traffic deaths. Therefore, a common ground in equity must be pursued by relevant stakeholders so that the benefits of preventing police strikes such as road mortality burden reduction, outweigh the risks.

## CONCLUSION

Given the knowledge gap, we opine that strengthening injury surveillance to understand the magnitude of road traffic deaths during police strikes is needed. Additionally, consistent with the UN Global Road Safety resolution, and a submission made by The Lancet in the light of the second Decade of Action for Road Safety, in which both documents identified police, health and transport sectors as active stakeholders in road safety [2,15], it is therefore necessary that advocacy efforts to prevent any police strikes should not be limited to only the police, justice, and government sectors but other relevant systems such as health and transport also have huge role to play, given its potential negative effect on road safety. Together, these systems will provide evidence-based recommendations (eg, improved social welfare, law reforms) that would be beneficial to prevent police strikes and in turn ensure continued access of road traffic injury victims to efficient life-saving pre-hospital trauma care interventions.
